# Use of the landfill water pollution index (LWPI) for groundwater quality assessment near the landfill sites

**DOI:** 10.1007/s11356-016-7622-0

**Published:** 2016-09-17

**Authors:** Izabela A. Talalaj, Pawel Biedka

**Affiliations:** Department of Environmental Engineering Systems, Bialystok University of Technology, Wiejska 45A Street, 15-351 Bialystok, Poland

**Keywords:** Groundwater quality, Indices, Landfill impact, Leachate, Pollution

## Abstract

The purpose of the paper is to assess the groundwater quality near the landfill sites using landfill water pollution index (LWPI). In order to investigate the scale of groundwater contamination, three landfills (E, H and S) in different stages of their operation were taken into analysis. Samples of groundwater in the vicinity of studied landfills were collected four times each year in the period from 2004 to 2014. A total of over 300 groundwater samples were analysed for pH, EC, PAH, TOC, Cr, Hg, Zn, Pb, Cd, Cu, as required by the UE legal acts for landfill monitoring system. The calculated values of the LWPI allowed the quantification of the overall water quality near the landfill sites. The obtained results indicated that the most negative impact on groundwater quality is observed near the old Landfill H. Improper location of piezometer at the Landfill S favoured infiltration of run-off from road pavement into the soil-water environment. Deep deposition of the groundwater level at Landfill S area reduced the landfill impact on the water quality. Conducted analyses revealed that the LWPI can be used for evaluation of water pollution near a landfill, for assessment of the variability of water pollution with time and for comparison of water quality from different piezometers, landfills or time periods. The applied WQI (Water Quality Index) can also be an important information tool for landfill policy makers and the public about the groundwater pollution threat from landfill.

## Introduction

Despite different possibilities of municipal waste treatment, including recycling, composting and incineration, municipal landfills are still a common way of waste disposal in many regions of the world. Data from 2013 show that in 14 countries of the European Union, the share of landfilling is over 50 % and in 6 of these countries even over 75 % (Greece, Croatia, Cyprus, Latvia, Malta, Romania) (Eurostat 2015). In USA, about 135 million tons of solid waste (53.8 %) were discarded in landfills in 2012 (USEPA 2012). In most low to medium income developing countries, almost 100 % of municipal solid waste generated goes to landfills (Longe and Balogun 2010).

Landfills pose serious threat to the quality of environment if they are incorrectly secured and improperly operated. The scale of this threat depends on the composition and quantity of leachate, time of landfill exploitation, distance of a landfill from a plant, soil and water environment, etc. Groundwater contamination is a major concern in landfill operations because of the pollution effect of landfill leachate and its potential health risks (Bhalla et al. 2012). Therefore, the migration of landfill leachate into surface or groundwater is considered to be a serious environmental problem at both uncontrolled and engineered municipal landfill sites (Ettler et al. 2008). The environmental impact of the landfill leakage on groundwater quality has been noticed several times regardless of an ideal site selection and introduction of geomembrane layers. Municipal landfill leachate is highly concentrated complex effluents, which contain dissolved organic matter, inorganic compounds, heavy metals and xenobiotic organic substances (Christensen et al., 2001). Therefore, evaluation of a potential risk associated with groundwater contamination due to landfills is of great importance.

To evaluate the groundwater contamination, WHO standards for drinking water are usually used (Longe and Balogun 2010; Vasanthavigar et al. 2010; Gibrilla et al. 2011) however, they are not always adequate for potentially strongly contaminated groundwater in the vicinity of a landfill. Besides, a large number of separate parameters do not easily provide a general view of the level of groundwater contamination (Backman et al. 1998). Several researchers have proposed different methods and indices for evaluation of groundwater quality data (Alobaidy et al. 2010; Gibrilla et al. 2011). The most popular is Horton’s water quality index (WQI), which is defined as a rating, reflecting the composite influence of different water quality parameters (Shivasharanappa et al. 2011). Horton selected 10 most commonly measured water quality variables for his index, including dissolved oxygen (DO), pH, total coliform bacteria, specific conductance, alkalinity, chloride, biological oxygen demand (BOD), chemical oxygen demand (COD), temperature and nitrogen (Water Quality Indices 2012). WQI is calculated from the point of view of the suitability of groundwater for human consumption and does not take into account any toxic chemicals, which can appear in groundwater near a landfill site. Brown et al. (1970) developed a water quality index similar in structure to Horton’s index but with much greater rigour in selecting parameters. Brown’s index represents a general water quality; however, it does not recognize and incorporate specific water function (for example drinking water supply) or water quality (agriculture, industrial, landfill area, etc.) (Water Quality Indices 2012). A multiplicative water quality index was developed by Dinius (1987). The index included 12 pollutants—DO BOD, coliform count, *E. coli*, pH, alkalinity, hardness, chloride, specific conductivity, temperature, colour and nitrate—for six water uses—public water supply, recreation, fish, shellfish, agriculture and industry. It does not include specific water quality of landfill areas. Another index, developed by The Oregon Department of Environmental Quality, The Oregon water quality index (OWQI) is integrating measurements of eight water quality variables (temperature, DO, BOD, pH, ammonium and nitrate nitrogen, total phosphates, total solids and faecal coliform). The OWQI helps to evaluate the effectiveness of water quality management activities but is not adopted for assessment in a strongly polluted landfill water (Cude 2001, 2002).

Although all of the above mentioned methods have their advantages, they are not proper for assessment of strongly polluted groundwater in the vicinity of landfills. Moreover, different parameters are taken into consideration in the proposed methods which make it difficult to compare the obtained results. What is more, a large number of separate parameters do not easily provide a general view of the level of groundwater contamination by landfill leachate. In conclusion, there is a lack of simple method, which can be easily used for groundwater assessment near landfill sites.

To remedy this situation, a landfill water pollution index (LWPI) was proposed by Talalaj (2014). It takes into account 10 parameters which, according to European Union regulation, should be monitored obligatorily in groundwater near the landfill, both during its exploitation and after the closure. So such data are available for all landfill managers, and they are easily accessible in environmental protection agencies. Moreover, this set of parameters includes most of variables used for calculation of the leachate pollution index (LPI), proposed by Kumar and Alappat (2005).

The aim of this study is to evaluate the applicability of LWPI in three landfill areas of varied hydrologic circumstances and with different time of landfill exploitation.

## Materials and methods

### Landfill sites

The groundwater samples were collected in the vicinity of three municipal landfills (Landfill H, Landfill E, Landfill S) localized in different regions of Poland.

#### Landfill H

**Fig. 1:**
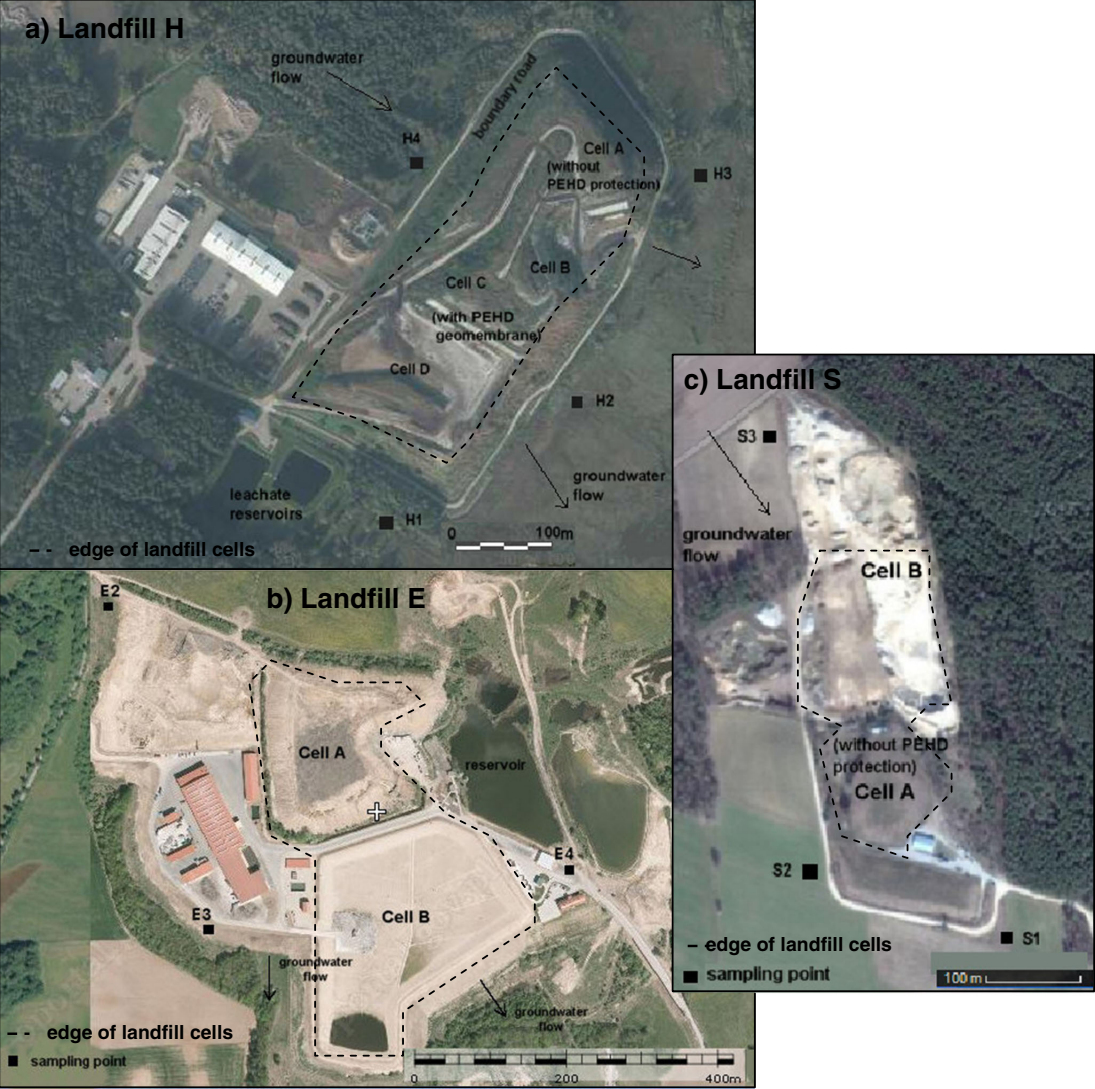
Localization of Landfill H, Landfill E, Landfill S and sampling points

The Landfill H is the biggest landfill in Podlasie Province in the eastern part of Poland. It has been operated since 1981 and covers an area of 40 ha (Fig. 1a). According to the instruction for the landfill operation, only municipal waste (apart from fluid waste, sewage sludge, hazardous substances, radioactive and toxic waste) may be deposited on Landfill H. The following kinds of vehicles are used for site operation: waste trucks, tanker trucks, compactor, rubber-tired tractor, bulldozers and pickup trucks. The total amount of solid waste deposited in the landfill till the end of 2010 was estimated at 308,000 m^3^. The Landfill H consists of four sections (cells), from which the oldest one—the Cell A—closed in 2001 is not equipped with a lining system. To protect groundwater from leachate infiltration, it was sealed with a 50-cm clay layer. The rest of the cells are lined at the bottom with impermeable 2 mm HDPE (high-density poliethylen) geomembrane. In these cells, leachates are collected by perforated pipes on top of the liner and they are pumped out of site to a retention reservoir. Then, they are transported outside the Landfill H, to a municipal sewage treatment plant. The leachate amount is about 25,000 m^3^ annually.

The analysed area is covered by a sand formation, which is underlain by a complex of boulder clay. The first aquifer is composed of sands with uniform granulation (ϕ = 0.05 ÷ 0.20 mm) and the infiltration coefficient *k* = 10^−4^ m/s ÷ 10^−5^ m/s. The thickness of this strata ranges from 0.7 to 12.0 m. For these parameters and low hydraulic gradient values, groundwater flow is of 10 to 50 m/year. A free groundwater table lays 0.95 – 5.4 m below the land surface (139.0 to 142.0 m above sea level). The landfill is under-washed on the west side by groundwater that flows in the northeast, southeast and in eastern directions.

Climate in the area of Landfill H is continental with an average rainfall of 550 mm and evaporation of 450 mm. Approximately, 40 % of the rainfall occurs from June to September—this can lead to increased infiltration in waste body and release of contaminants to the surrounding low-lying areas. Twenty-two percent of the rainfall occurs in autumn season, 17 %—in winter and 21 %—in spring. The average annual temperature is about 7 °C.

#### Landfill E

The Landfill E is situated in the Warminsko-Mazurskie Province in the northeastern Poland. It started its operation in 1983. The landfill covers an area of 25 ha, almost 150,000 m^3^ of waste is deposited on Landfill E per year. The Landfill E consists of two cells: Cell A and Cell B (Fig. 1b). The Cell A is sealed with a natural 31-m clay substrate functioning as a geological barrier. In order to protect the soil-water environment, a circumferential ditch to prevent contaminated water from getting out of the landfill area was constructed. At the beginning of 2010, the operation of the Cell A was finished. It is estimated that in the closed quarter (Cell A), 550,000 Mg of waste was deposited. The new Cell B started its operation in 2012. A high-density 2-mm polyethylene geomembrane (HDPE) was used in the barrier system to prevent leachate infiltration into the soil-water environment. There is also a leachate drainage system placed on the bottom of this cell. The leachate from both Cell A and Cell B are collected and transported to an equalization tank. Then, they are directed to the on-site purification plant (reverse osmosis system). The concentrate from reverse osmosis is recirculated on a new Cell B. The yearly amount of generated leachate from two cells is about 21,000 m^3^.

The landfill area is situated on glacial clays covered with kame hills. Glacial clays lie at a depth of 31–56 m. Two water-bearing layers were found on area of Landfill E:

- inter-clay waters—found out in the E4 piezometer—the aquiferous layer is made of sand lenses occurring at various depths. A complex of glacial clays of a thickness above 50 m makes the isolation of the aquiferous layer. Water runs off in the SE and S direction.
- intermoraine waters—found out in the E2 and E3 piezometers—fine and medium sands constitute the aquiferous layer. The isolation of this layer is a complex of boulder clays; the thickness of which does not exceed 49.5 m. The run-off direction of waters is S. Piezometr E2 is situated at the inflow of groundwater to the landfill, and its indications were taken as the pollution background.

The average rainfall in this region is about 650 mm and evaporation—430 mm annually. Approximately, 40 % of the rainfall occurs in the summer season, 23 %—in autumn season, 15 %—in winter and 22 %—in spring. The average annual temperature is about 8 °C.

#### Landfill S

The Landfill S is located in the Podlaskie Province in the northeastern Poland. The landfill site, with the total surface area of 4.0 ha, has been operated since 2003. It consists of two waste cells named A and B, with a total area of 2.6 ha (Fig. 1c). The Cell A has not been sealed with geomembrane, and the only barrier that protects groundwater against contamination is a 50-cm layer of clay. It was closed in 2009 after 7 years of exploitation. In 2010, the Cell B was constructed with 2-mm geomembrane (HDPE) liner system and a leachate collection systems. Generated leachate from this cell was collected by perforated pipes, temporarily stored and transported out of the landfill for off-site treating. The capacity of two cells is estimated at 59,000 m^3^.

The analysed area to a depth of at least 80 m constitutes quaternary formations consisting of clay. The near-surface layer constitutes medium-grain sands. The groundwater level is closely related to the relief, which slopes in the south and southeast direction. The depth of the groundwater level is from 8 to 20 m under the ground surface. Waters on this level can be highly exposed to a physiochemical and bacteriological contamination.

The climate in this region is continental with close to 70 % of rainfall occurring between May and September. The lowest rainfall is observed in January and March. The average yearly precipitation is about 590 mm per year, average temperature is 6.2 °C and evaporation is 450 mm.

## Methodology

### Landfill H

For the evaluation of groundwater contamination, samples of groundwater were taken since 2004 till 2014, four times a year. The groundwater samples were collected from three observation points (piezometers)—H1, H2, H3—situated at the groundwater outflow from the landfill and one observation point—H4—situated at the inflow of groundwater to the landfill. The H4 was out of the range of the landfill influence and was taken as a pollution background. In order to obtain reliable results, sampling under specific atmospheric conditions, e.g. after periods of intensive precipitation or following long-lasting periods without precipitation (droughts), was avoided. On the whole, during the research, 166 samples (over forty from each sampling point) were taken.

### Landfill E

Groundwater and surface water data were collected from spring 2005 till spring 2012, i.e. 3 years after closing of the Cell A. Sampling was done four times a year in each season. Samples were taken from one background E2 piezometer—localized on the groundwater inflow and two piezometers E3 and E4 situated at the groundwater outflow from the landfill. On the whole, during the research, 108 samples (36 from each piezometer) were taken.

### Landfill S

Samples of groundwater were taken three times a year, since 2010 till 2014 i.e. for the next 5 years after Cell A closure. In 2013 and 2014, sampling was made only twice, in II and IV quarters of the year. A data from one sampling in 2004 was also used in the study. Samples were collected from two observation points (piezometers)—S1 and S2—situated at the groundwater outflow from the landfill and one observation point—S3—situated at inflow of groundwater to the landfill (background). On the whole, during the research, 42 samples (14 from each sampling point) were taken.

The summary characterization of sampling points is presented in Table 1.

**Table 1:** Groundwater sampling localization and characterization

Sample	Location characterization	Distance from the edge of landfill cell	Depth to the water level
		(m)	(m)
Landfill H			
H4	Inflow (background)	65	3.0
H1	Outflow	90	2.5
H2	Outflow	70	1.5
H3	Outflow	60	2.9
Landfill E			
E2	Inflow (background)	250	28
E3	Outflow	180	34
E4	Outflow	250	14
Landfill S			
S3	Inflow (background)	130	18
S1	Outflow	150	8
S2	Outflow	55	7

The groundwater samples were analysed—according to the Polish Regulatory of Landfill Monitoring (Regulation of Minister of Environment concerning the landfills 2013)—for pH, electroconductivity (EC), polycyclic aromatic hydrocarbons (PAH), total organic carbon (TOC) and six heavy metals: Cr, Hg, Zn, Pb, Cd, Cu. Determination was carried out in accredited laboratory, according to the Polish Standards. The pH was measured the same day as the samples were collected, using potentiometric method (according to PN90/C-04,540-01), EC—using conductivity method (PN-EN27888:1999). TOC was measured with infrared spectrometry method (PN-C-04,633-3:1994), PAH—with HPLC method with fluorescence detection (PB-05-78/PAI 2:25.06.2007). The heavy metals—except for Hg—were analysed by atomic emission spectrophotometry ICP-OES (PN-EN ISO 11885:2009), and Hg was determined by atomic absorption spectrophotometry (PB-IN 4:04.11.2010). Obtained results were the mean value of three determinations carried out simultaneously.

The landfill water pollution index (LWPI) was used to estimate the landfill influence on water quality (Talalaj, 2014):

1 $$ \mathrm{LWPI}=\frac{{\displaystyle \sum_{i=1}^n\Big(}{w}_i\cdot {S}_i\Big)}{{\displaystyle \sum_{i=1}^n{w}_i}} $$

where LWPI is the quality index for groundwater impacted by a landfill, *w*_*i*_ is the weight of the *i*—the pollutant variable and *n*—the number of groundwater pollutants.

The *S*_*i*_ is calculated from the equation:

2 $$ {S}_i={C}_p/{C}_b $$

where *C*_*p*_ is the concentration of the *i*—the parameter in each of a groundwater outflow (polluted) sample, and the *C*_*b*_ is the concentration of the *i*—the parameter in an inflow (background) groundwater sample. Weight values were assigned to each of the analysed parameter, reflecting the influence of each parameter on the groundwater quality. A full method description is given by Talalaj (2014).

Data analysis included mean, minimum, maximum, standard deviation, trend line of chemical variation vs. time to assess the direction of changes and analysis of variance to determine the effect of season and landfill localization on the groundwater quality. Due to large number of samples, specific actions were undertaken during data analysing to ensure quality control i.e. checking for completeness across category and years, making sure data line up are in proper columns and there are no missing, impossible or anomalous values, performing statistical summaries and looking for outliers, or extreme values, to identify possible data ‘contamination’.

## Result and discussion

### Physicochemical analysis

Analytical results of physicochemical characteristics of groundwater inflowing and outflowing from analysed landfill and its basic statistics are shown in Table 2. Obtained results were also compared to the WHO and Polish standards for drinking water quality (Regulation of Minister of Health concerning quality of water for human consumption 2007; WHO 2011).

**Table 2:** Statistical analysis of groundwater characterization

Landfill H									
	Groundwater inflow (background)				Groundwater outflow (polluted)				WHO/PL standards for drinking water quality
Parameter	*n* = 44				*n* = 122				
	Mean	Min	Max	St. dev.	Mean	Min	Max	St. dev.	
pH	6.82	5.70	7.80	0.483	6.97	4.70	7.84	0.541	*6.5–8.5*
EC	0.5	0.09	1.64	0.429	4.237	0.00	14.40	3.944	*−/−*
Cd	0.004	0.000	0.063	0.012	0.005	0.000	0.070	0.014	*0.003/0.005*
Pb	0.024	0.001	0.094	0.031	0.025	0.001	0.150	0.035	*0.01/0.025*
Zn	0.070	0.000	0.640	0.121	0.149	0.000	8.893	0.851	*−/5.0*
Cu	0.014	0.001	0.154	0.031	0.038	0.001	0.850	0.085	*2.0/2.0*
TOC	15.57	0.50	51.10	13.74	118.11	10.92	616.6	126.69	*−/−*
PAH	2.395	0.000	47.960	8.605	2.166	0.000	96.020	9.606	*−/0.1*
Cr	0.015	0.000	0.116	0.029	0.019	0.000	0.152	0.036	*0.005/0.05*
Hg	0.001	0.000	0.010	0.001	0.001	0.000	0.012	0.002	*0.006/0.001*
Landfill E									
Parameter	Groundwater inflow (background)				Groundwater outflow (polluted)				WHO/PL standards for drinking water quality
	*n* = 36				*n* = 72				
	Mean	Min	Max	St. dev.	Mean	Min	Max	St. dev.	
pH	7.24	6.71	7.73	0.221	7.21	6.58	7.77	0.227	*−/−*
EC	0.688	0.383	1.288	0.140	0.905	0.360	1.715	0.277	*−/−*
Cd	0.001	0.000	0.001	0.000	0.001	0.000	0.003	0.000	*0.003/0.005*
Pb	0.004	0.001	0.010	0.002	0.004	0.001	0.021	0.003	*0.01/0.025*
Zn	0.032	0.001	0.100	0.023	0.028	0.005	0.091	0.018	*−/5.0*
Cu	0.005	0.001	0.014	0.004	0.004	0.001	0.010	0.004	*2.0/2.0*
TOC	2.044	0.500	9.860	2.020	2.628	0.001	13.600	2.380	*−/−*
PAH	0.022	0.003	0.100	0.023	0.093	0.003	2.876	0.413	*−/0.1*
Cr	0.004	0.000	0.010	0.004	0.004	0.000	0.010	0.004	*0.005/0.05*
Hg	0.000	0.000	0.000	0.000	0.000	0.000	0.000	0.000	*0.006/0.001*
Landfill S									
Parameter	Groundwater inflow (background)				Groundwater outflow (polluted)				WHO/PL standards for drinking water quality
	*n* = 14				*n* = 28				
	Mean	Min	Max	St. dev.	Mean	Min	Max	St. dev.	
pH	7.68	7.30	9.26	0.470	7.23	6.86	9.30	0.546	*−/−*
EC	0.52	0.31	0.61	0.081	1.48	0.00	2.23	0.638	*−/−*
Cd	0.019	0.000	0.030	0.015	0.019	0.000	0.030	0.014	*0.003/0.005*
Pb	0.088	0.008	0.120	0.029	0.075	0.007	0.100	0.037	*0.01/0.025*
Zn	0.145	0.050	0.670	0.183	0.130	0.050	0.560	0.122	*−/5.0*
Cu	0.038	0.002	0.050	0.017	0.038	0.002	0.060	0.019	*2.0/2.0*
TOC	2.83	0.00	12.00	3.233	4.97	0.00	13.00	4.116	*−/−*
PAH	1.19	0.000	8.900	2.557	6.59	0.00	140.00	26.328	*−/0.1*
Cr	3.574	0.002	10.000	4.970	3.575	0.002	10.000	4.877	*0.005/0.05*
Hg	0.000	0.000	0.001	0.000	0.000	0.000	0.001	0.000	*0.006/0.001*

All in mg/l, expect EC in miliS/cm, PAH in μg/l and pH

The pH values for all groundwater samples are within the range of WHO and Polish standards (Regulation of Minister of Health concerning quality of water for human consumption 2007; WHO 2011). The average range of pH in groundwater inflow to the Landfill H was close to neutral—6.82. The observed pH in groundwater below the landfill was higher and reaches the value of 6.97. Increase of pH value in groundwater outflowing from the landfill was caused by a long time of landfill exploitation (above 30 years) and generation of stabilized and matured leachate with pH above 7.0.

The opposite situation is noted in Landfill S and Landfill E. The average pH in outflowing groundwater (pH = 7.23 for Landfill S and 7.21 for Landfill E) was lower than that in background (pH = 7.68 for Landfill S and 7.24 for Landfill E). This points that groundwater is acidified by a low-pH leachate from analysed landfills. The results indicate anaerobic or methanogenic fermentation stage of the leachate. This stage is usually characterized by production of volatile fatty acids and high partial pressure of carbon dioxide with a pH range of 6 to 8. (Kjeldsen et al. 2002; Longe and Balogun 2010).

The EC value is the indicator of dissolved inorganic ions in groundwater (Mor et al. 2006; Kale et al. 2010). The average EC value in Landfill H was 0.5 miliS/cm in background and 4.24 miliS/cm in outflowing groundwater. In Landfill E, the value of EC in groundwater outflowing from the landfill was 0.9 miliS/cm while in background water 0.7 miliS/cm. The EC in Landfill S was 0.5 miliS/cm in groundwater inflowing into landfill and 1.48 miliS/cm in outflowing water. The standard deviation of EC value in landfills H, E and S were 3.94, 0.28, 0.6, respectively, pointing to the big differentiation of EC values, mainly in the old Landfill H.

Chemical analysis of heavy metals shows that the metal concentration in groundwater near the Landfill H can be ranged as follow: Zn > Pb > Cr > Cu > Cr > Cd > Hg—in groundwater inflow and Zn > Cu > Pb > Cr > Cd > Hg—in groundwater outflow. The average values of analysed heavy metals in background and outflowing groundwater from Landfill H did not exceed the Polish standards for drinking water quality (Table 2). The biggest difference was observed in cases of Zn and Cu; the concentration of which in outflowing groundwater were 0.149 and 0.038 mg/l, respectively, i.e. two times more than that in background water. The higher concentration of Zn may be due to the presence of zinc-based waste like zinc-plated materials, fertilizers and cement (Singh et al. 2008). Packaging fertilizers—although they should be extracted as hazardous waste—are a common component of municipal waste coming from the surrounding rural areas. Besides, a substantial part of Zn seems to be related to inorganic colloids, which makes Zn more mobile than other metals (Christensen et al. 2001). A source of copper in groundwater can be plant protection products, fertilizers or copper salts used for biofilm destruction in water pipes (Christensen et al. 2001).

The concentration of heavy metals in the groundwater near Landfill E was relatively low and does not exceed WHO and PL standards for drinking water quality. The amount of analysed metals both in outflowing and inflowing groundwaters can be set in the following order: Zn > Cu > Cr > Pb > Cd > Hg. The average concentration of Cu, Cr, Pb, Cd and Hg is the same in the upstream and downstream water. It means that 50-m clay layer below the landfill bottom is a sufficient barrier against heavy metal migration into groundwater and enables a sorption of heavy metals on organic colloids. Heavy metals in leachate plume are also attenuated by precipitation and dilution processes (Christensen et al. 2001). The higher concentration of Zn (0.032 mg/l) in groundwater inflowing to the landfill suggests that upstream water is already polluted probably by local pollution sources. The Zn concentration in downstream water is 0.028 mg/l.

The concentration of analysed heavy metals both in inflowing and outflowing groundwater of Landfill S can be ordered in following way: Cr > Zn > Pb > Cu > Cd > Hg. Water flowing into the landfill is already contaminated, and the concentration of Cd, Pb and Cr in background exceeds the WHO and Polish standards (Regulation of Minister of Health concerning quality of water for human consumption 2007; WHO 2011) and reaches the values 0.019, 0.088 and 3.574 mg/l, respectively. Cd and Cr concentrations in groundwater flowing out of the landfill do not change and stay at the same level. Pb concentration decreases to the value of 0.075, which, however, is still above permissible limits. Concentrations of Cu and Hg in inflowing and outflowing groundwater near the Landfill S were the same and reached the value of 0.038 mg/l for Cu and 0.000 mg/l for Hg, being below permissible limits of WHO/PL. The value of Zn (0.130 mg/l) in groundwater outflowing from the landfill was lower than that in water coming to landfill (0.145 mg/l). Obtained results show that landfill is not a source of heavy metals in groundwater. Land use on the adjacent area indicates that these metals may come from crop protection, chemicals and fertilizers.

The TOC and PAH are indicators of dissolved organic matter in analysed groundwater. These two parameters have the highest value of standard deviation in all analysed landfills. It indicates a high variability of these parameters with time. At Landfill H, the TOC value in outflowing groundwater was 118.1 mg/l and was eight times higher than that in background water (15.6 mg/l of TOC). The value of TOC in Landfill E in background was 2.044 mg/l, while 2.628 mg/l in outflowing water. Similar values of TOC were observed at Landfill S, and the value of TOC in inflowing and outflowing groundwater was 2.8 and 4.97 mg/l, respectively.

The value of PAH in background water of Landfill H was 2.4 μg/l and was higher than that in outflowing water with 2.17 μg/l of PAH. High concentration of PAH in groundwater can be the result of washing out from the road pavement contamination coming from car exhausts, from wearing off car tire and from asphalt rich in hydrocarbon fractions, which can all next infiltrate to groundwater along with surface run-offs. Localization of the H4 piezometer (background) by the road surrounding the landfill points to the possibility of water contamination in this piezometer with PAH compounds.

At Landfill E, the PAH concentration in groundwater inflow was 0.022 μg/l. Despite the fact that concentration of PAH in groundwater outflow increased over four times to the value of 0.093 μg/l, it does not exceed the permission level for drinking water quality for Poland.

The value of PAH in Landfill S was 1.19 μg/l in background and 6.6 μg/l in outflowing groundwater. According to Christensen et al. (2001), the concentration of PAH in landfill leachate plume is expected to decrease with time, depending for each compound on its degradation in landfill and its volatilization with the landfill gas. However, PAH is not very extensively attenuated by sorption onto aquifer material what could be seen on analysed landfills. Some recent reports show that they can be degradable in the strongly anaerobic environment in leachate plume (Christensen et al. 2001).

### Landfill pollution index (LWPI) analysis

Table 3 shows the calculations for LWPI for three analysed landfills:

over 30-year-old Landfill H with closed and unsealed Cell A and sealed Cells B, C, D;

over 20-year-old Landfill E with closed and unsealed Cell A and one sealed Cell B;

over 10-year-old Landfill S with closed and unsealed Cell A and new sealed Cell B.

**Table 3:** Statistical analysis of LWPI values

Piezometer	Mean	Min	Max	Stand. dev	*N*
Landfill H					
H1	4.03	0.52	17.30	3.732	44
H2	8.89	0.60	46.12	10.493	37
H3	10.38	0.70	98.25	18.535	41
Landfill E					
E3	1.11	0.70	2.53	0.362	36
E4	6.62	0.78	116.04	21.871	36
Landfill S					
S1	13.79	0.70	169.0	44.706	14
S2	1.737	0.80	5.20	1.116	14

Interpretation (Talalaj 2014):

LWPI ≤1 water without landfill impact

1 < LWPI ≤2 moderately polluted water due to a small landfill impact

2 < LWPI ≤5 poor water with high visible landfill impact

LWPI >5 strongly polluted water under an evidently very high landfill impact

**Fig. 2:**
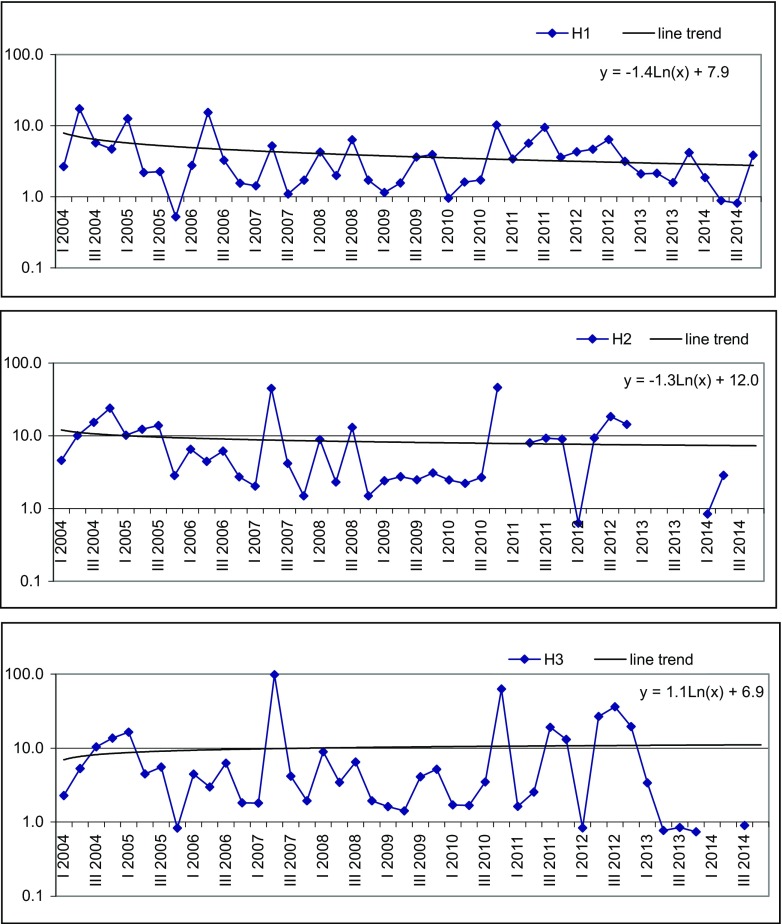
Variability of LWPI value with time at Landfill H

**Fig. 3:**
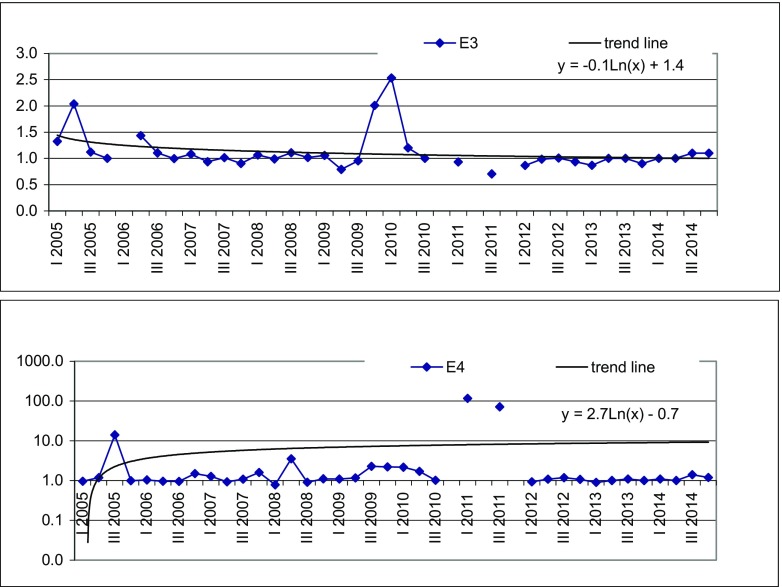
Variability of LWPI value with time at Landfill E

**Fig. 4:**
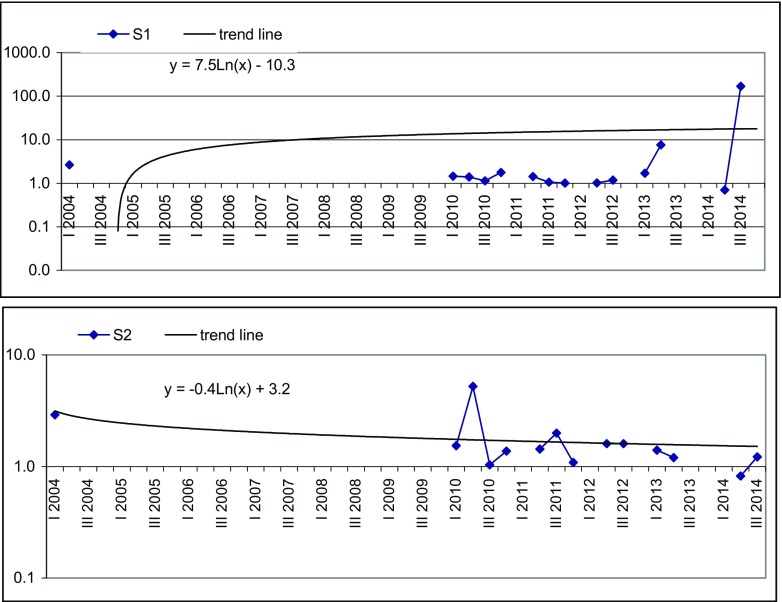
Variability of LWPI value with time at Landfill S

Figures 2, 3 and 4 present changes of LWPI value in analysed piezometers with time. Due to the wide range of LWPI values, a logarithmic scale for *Y*-axis was used in the graphs (except the graph for E3 piezometer) in order to better illustrate the obtained results.

At Landfill H, the average value of LWPI in H1 piezometer was 4.03. The LWPI was the highest at the start of observation period—in II quarter of 2004—reaching the value of 17.3 due to high TOC concentration (98.3 mg/l). The increase of LWPI was also observed in I quarter of 2005, II quarter of 2006, IV quarter of 2010 and III quarter of 2011, and it was caused mainly by elevated value of TOC. According to Christensen et al. (2001), organic carbon—especially its dissolved forms—practically moves with water or is just slightly retarded. Thus, sorption cannot be viewed as a significant attenuation mechanism for dissolved organic carbon in aquifers, however, it may play a role in attenuation of insoluble fraction of organic carbon. The plotted line trend with formula *y* =  − 1.4 ⋅  ln (*x*) + 7.9 indicates that the value of LWPI decreases with time (Fig.2). Nevertheless, groundwater in H1 is classified as a poor one with a visible landfill impact (Table 3).

The average value of LWPI in H2 piezometer was 8.9. The maximum of 46.1 was observed in IV quarter of 2010 and was caused by high value of TOC (143 mg/l). Similar LWPI value of 45 was noted in II quarter of 2007, and the reason of this increase was high concentration of TOC (103.7 mg/l), PAH (1.18 μg/l) and Zn (4.0 mg/l). Observed results support the low sorption of dissolved organic carbon to any substantial degree onto aquifer material. Increase of PAH and Zn was caused by surface run-off rather than landfill leachate since heavy metals, such as Zn in landfill leachate plume, are controlled by sorption, possibly precipitation and complexation, and do not constitute a groundwater pollution problem at landfills (Christensen et al. 2001; Mor et al. 2006). The formula of line trend *y* =  − 1.3 ⋅  ln (*x*) + 12.0 indicates a decrease in LWPI value with time (Fig. 2). The value of LWPI classified groundwater in H2 piezometer as strongly polluted with high landfill impact (Table 3).

The LWPI value in H3 piezometer was 10.4. The highest value of 98.2 was noted in II quarter of year 2007 and—as in the case of H3 piezometer—was caused by high concentration of TOC, PAH and Zn. The line trend formula *y* = 1.1 ⋅  ln (*x*) + 12.0 demonstrates a slight increase of LWPI (Fig. 2). Standard deviation for LWPI value of the three piezometers H1, H2 and H3 is the highest in the last one, pointing a variation of LWPI during observation period. From Fig. 1, one can see that piezometer H3 is located near closed and unsealed Cell A. A high range of LWPI in this piezometer classifies the water as strongly polluted with very high landfill impact and indicates that Cell A still poses a threat for groundwater in the immediate vicinity of Landfill H.

The average LWPI value in E3 piezometer at Landfill E was 1.11 which means that groundwater is moderately polluted with small landfill impact (Table 3). A low degree of groundwater contamination results from the geological structure of the ground under the landfill. The clay layer exceeding at some places even 50 m is a physical barrier restricting the migration of contaminants. It also provides good conditions for sorption of pollutants. According to Xie et al. (2009) and Zhan et al. (2014), soil particles play an important role, especially in heavy metal adsorption and COD attenuation. The maximum LWPI of 2.53 was noted in I quarter 2010 due to TOC concentration (3.5 mg/l), seven times higher than that in background and Cd concentration (0.0012 mg/l) and 10 times higher than that in the control piezometer. Also, concentration of Cd was higher than in background and it was still below WHO and Polish standards for drinking water quality. As most heavy metals, Cd is subjected to strong attenuation by sorption and precipitation in the leachate plume as well as sorption on clay constituting landfill substrate (Singh et al. 2008). The line trend formula *y* =  − 0.1 ⋅  ln (*x*) + 1.4 indicates a slight decrease of LWPI with time (Fig. 3).

The average value of LWPI in E4 piezometer was 6.62 (Table 3). The two peaks of LWPI were noted in I and III quarters of 2011 and were 116 and 71.5, respectively. They affected largely the average value, which without two above mentioned peaks could be 1.64. The high value of LWPI was caused by PAH concentration, which in I quarter of 2011 was over 1000 times higher than in background and in III quarter of 2011—over 700 times higher. The E4 piezometer inlet is situated on the road to the landfill, so it is possible that surface run-offs from the road and surrounding area get into the piezometer and reach groundwater. It is worth noticing that PAH concentration in this piezometer was higher during the Cell B operation, which means that fuel spills from vehicles arriving at the landfill could have been the source of this contamination. The trend line with formula *y* = 2.7 ⋅  ln (*x*) − 0.7 indicates the increase of LWPI value with time; however excluding two incidental peaks, its formula of *y* =  − 0.7 ⋅  ln (*x*) + 3.5 reflects better than the actual situation.

The LWPI values at Landfill S are presented in Fig. 4. Since the data for all quarters of the year were not available, the LWPI has been calculated on the basis of available data. The average LWPI value in S1 piezometer was 13.8 with a maximum of 169 in III quarter of 2014, caused by high concentration of PAH, which in the analysed piezometer was 1000 times higher than in background water. PAHs are one of the most widespread organic pollutants. In addition to their presence in fossil fuels, they are also formed by incomplete combustion of carbon-containing fuels such as wood, coal, diesel, biomass, etc. (Tobiszewski and Namieśnik 2012). According to Cecinato et al. (2014), petrogenic sources and wood burning seemed to be the most important PAH sources in winter while in summer the contribution of street dust resuspension was important. The high standard deviation value of 447.7 confirms the big fluctuation of LWPI in piezometer S1. The trend line formula *y* = 7.5 ⋅  ln (*x*) − 10.3 suggests a successive—since 2010—increase of LWPI value in analysed piezometer (Fig. 4). The reason of this is both exploitation since 2010 of a new Cell B on landfill area and location of S1 piezometer direct on groundwater downgradients from unsealed Cell A. There is also the possibility of contamination of groundwater in S1 piezometer from surface run-off from the access road to the landfill. Location of S1 and shallow groundwater level favours potential contamination. A high PAH concentration at this control point confirms this hypothesis.

The average value of LWPI in S2 piezometer was 1.7, classifying groundwater as moderately polluted with a small landfill impact (Table 3). The maximum of LWPI of 5.20 was observed in II quarter 2010 and was caused by high TOC concentration of 140 mg/l. After this time, the LWPI decreased steadily as confirmed by the formula of line trend *y* =  − 0.4 ⋅  ln (*x*) + 3.1 (Fig. 4). In 2014, the LWPI value oscillated around 1 what suggests the improvement of groundwater quality in S2 piezometer but also—what is more probably—indicates a poor location of S2 piezometer behind the landfill leachate plume.

#### Seasonal and local differences of LWPI

In order to evaluate the change of LWPI in each season of the year and differentiation between the landfills, the variation analysis was done and results are presented in Table 4.

**Table 4:** Variance analysis of seasonal and local differentiation of LWPI

	SS effect	df effect	MS effect	SS error	df error	MS error	*F*	*p*
Results for seasonal differentiation								
LWPI	708.64	3	236.21	64,020.6	219	292.33	0.808	0.491
Results for site differentiation								
LWPI	692.47	2	346.23	64,036.8	220	291.08	1.189	0.306

*SS* sum square, *MS* mean square, *df* degrees of freedom, *F F* test value, *p* probability level

**Fig. 5:**
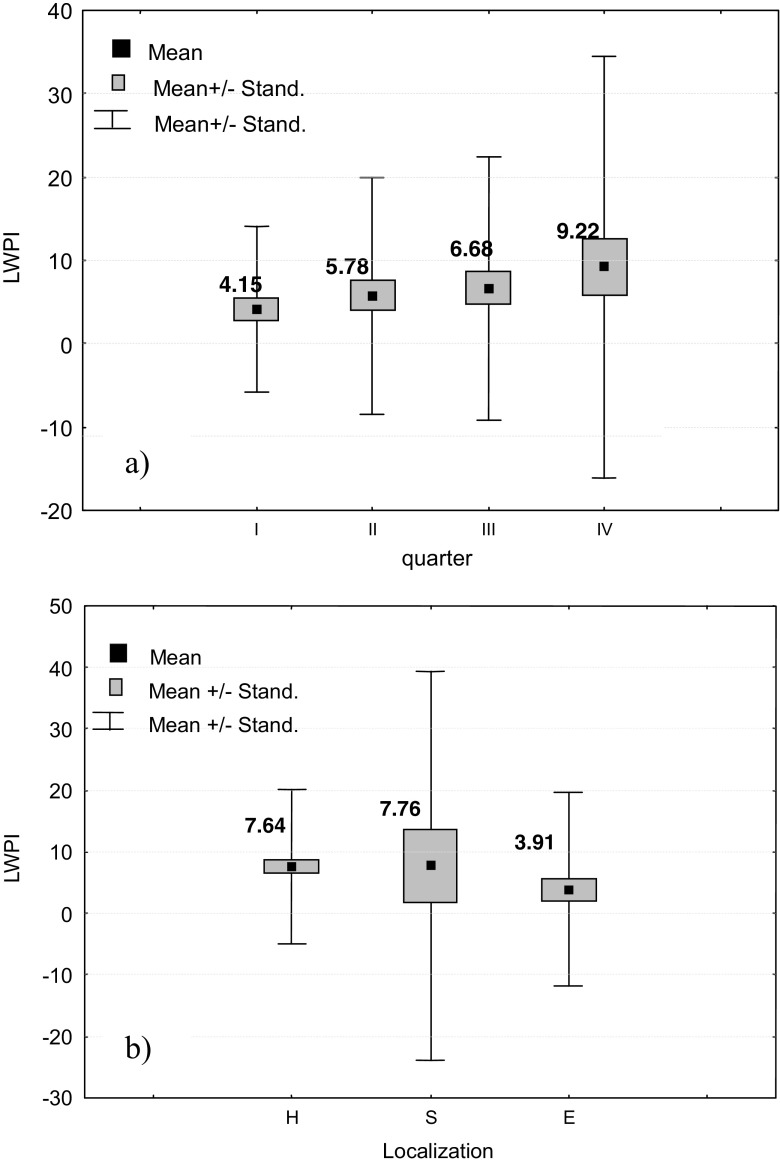
LWPI value depending on the season (a) and landfill location (b)

Results of analysis prove that observed seasonal differentiation of LWPI value on analysed landfills are not statistically significant (Table 4). From Fig. 5a, it is seen that on all analysed landfills, the LWPI value was the highest in the IV quarter, i.e. since October till December. An increased LWPI value results probably from a low precipitation and hence from lack of contamination dilution by rainfalls. According to Ettler et al. (2008), concentration of basic pollutants is generally higher in a dry season and decreases by simple dilution during precipitation events. The lowest LWPI value on analysed landfills was observed during snow-melt season and high precipitation, i.e. in I and II quarters of the year (Fig. 5a). Dilution by rainfall has also been observed by Durmusoglu and Yilmaz (2006) and Pinel-Raffaitin et al. (2006) at landfill sites which were analysed under their study. Another reason of a lower LWPI value is low temperature in this season of the year (4.7 °C), which limits the process of decay and slows down a number of physicochemical transformations occurring in water.

A differentiation of LWPI value in three analysed landfills was also analysed, and results were presented in Table 4 and Fig. 5.

The conducted analysis indicated that there were no statistical differences in the LWPI values on analysed landfills (Table 4). However, from Table 4 and Fig. 5b, it is seen that LWPI at Landfill H and Landfill S with shallow groundwater layer is higher than that at Landfill E with groundwater level over 50 m below the land surface. This indicates a high risk of contamination of shallow groundwater by landfill leachate and points effectiveness of clay underlying the E Landfill in pollutant retention. A 50-m subsoil level—acting as a buffer layer—reduced the pollution migration making a physical barrier from pollutants and enabling sorption, degradation and precipitation processes.

## Conclusion

Obtained results reveal that the quality of the groundwater near the three analysed landfill has been impacted by the landfill. The EC values, the TOC and PAH concentration in downstream groundwater were higher than those in the water inflowing to the landfills. The concentration of heavy metals and the pH usually remained at a similar level. The calculated LWPI values indicated that the most negative impact on groundwater quality is observed near the old Landfill H and Landfill S, operated for over 30 years. The average value of LWPI was 7.7 which indicates that contamination from the landfill is continuously washed away and gets into the soil-water environment. The high value of LWPI at Landfill H is also associated with shallow groundwater. A thin subsoil layer is not a sufficient barrier from pollutants and does not reduce the pollution migration into groundwater. At the Landfill S, exploitation of the new Cell B caused the deterioration of groundwater quality at the S1 piezometer. An increased value of the LWPI in this piezometer can also be the result of its location at the access road, which favours infiltration of run-off from road pavement into the soil-water environment. LWPI values were lower during periods of intensive precipitation than during periods of less intensive rainfalls; however, this observed differentiation was not significant from a statistical point of view. Deep deposition of the groundwater level reduces the landfill impact on the water quality. Over 50-m thick clay layer makes a barrier restricting the migration of contaminants and enables a sorption process. Average value of LWPI at this place was 3.9.

The results obtained in this study reveal that the landfill water pollution index can be used for groundwater quality assessment near a landfill site. It is an indicator which clearly and comprehensibly shows the degree of landfill impact on groundwater quality. By using it, you can assess the variability of results with time and compare the results obtained from different places/piezometers and time periods. The obtained results can be used for proper management of a landfill as well as for the protection of the groundwater quality. The LWPI can also serve as an information tool for landfill managers and the public about the groundwater quality and leachate pollution threat from the landfill.
